# Effects of Acetaminophen consumption in asthmatic children

**Published:** 2012-10-30

**Authors:** S Oshnouei, Sh Salarilak, A Khalkhali, M Karamyar, Mh Rahimi Rad, A Delpishe

**Affiliations:** 1 MSc student of Epidemiology, Epidemiology and Biostatistics Department, Faculty of Medicine, Urmia Medical University, Iran; 2 Associate Professor of Epidemiology, Public Health Department, Faculty of Medicine, Islamic Azad University, Tabriz Branch, Tabriz, Iran; 3 3Assistant Professor of Biostatistics, Epidemiology and Biostatistics Department, Faculty of Health, Urmia Medical University, Iran; 4 Associate Professor of Pediatrics, Pediatrics Department, Faculty of Medicine, Urmia Medical University, Iran; 5 Associate Professor of pulmonary Diseases, Faculty of Medicine, Urmia Medical University, Iran; 6 Associate Professor of Epidemiology, Public Health Department, Faculty of Health, Ilam Medical University, Iran

**Keywords:** Acetaminophen, Childhood asthma

## Abstract

**Background:**

Acetaminophen exposure might be associated with increasing risk of asthma prevalence and other atopic disorders over recent decades. The present study aimed to investigate the association between acetaminophen exposure and the risk of developing childhood asthma.

**Methods:**

A case - control study was undertaken between March and September 2010 in Urmia district north west of Iran. Subjects were children aged between 2 - 8 years old. Cases were asthmatic children diagnosed based on GINA criteria (n=207) and controls were children without asthma symptoms (n=414) using 1:2 sampling method. Cases and controls were matched for age and gender. Clinical data including Acetaminophen exposure was collected by a questionnaire which completed by interviewing with parents/ guardians.

**Results:**

Using Acetaminophen during the first year of life had no any effect on the risk of asthma (p=0.19), but amongst 2-8 years old children, this association was observed (p<0.001). There was also a doseresponseassociation between Acetaminophen consumption and risk of asthma (OR: 3.8; 95% CI; 2.15 6.59 for once per 2 to 3 month and OR: 4.2; 2.50 - 7.3 for at least one per month).

**Conclusions:**

Using Acetaminophen increases risk of asthma among 2 - 8 years old children. However stronger evidences are required to design evidence-based guidelines to reduce acetaminophen consumption following post - vaccination and other febrile disorders.

## Introduction

Asthma is one of the most common chronic diseases in childhood, affecting up to 300 million people in the world.(1) Using medicines in childhood has been considered risk factor in different communities. There is expanding information of an association between using Acetaminophen (Paracetamol) and increasing incidence of asthma during intrauterine (2,3) infancy (4,5), later childhood4 and adulthood periods (6,7). It has been reported that acetaminophen, increases asthma incidence rate up to 60%.(8) The main evidence for association between using of acetaminophen and the risk of childhood asthma develops from the International Study of Asthma and Allergies in Childhood, which included data from 72 centers in 31 countries worldwide (4). This association is constantly present in countries with different patterns of childhood febrile disorders (9-11). Countries with higher sale of Paracetamol or high Paracetamol use had a higher prevalence of asthma and other atopic disease (12).

Although the pathophysiology remains unclear, some important mechanisms have been reported (13). The frequent use of acetaminophen might increase risk of asthma and other allergic disorders. Acetaminophen also decreases levels of glutathione in the liver through a dose-dependent mechanism (14,15). High doses of Acetaminophen are cytotoxic for pneumocytes and maycause acute lung damage (16).

The present study aimed to investigate the association between acetaminophen exposure and the risk of developing childhood asthma in Urmia city, North West of Iran.

## Materials and Methods

A case - control study was conducted between March and September 2010 in Urmia city, North West of Iran. To reduce the risk of differential diagnosis between asthma and respiratory tract infections in small children, patients in the first 2 years of life excluded from case group.

Cases (n=207) were selected from asthmatic patients who their disease were confirmed based on the Global Strategy for Asthma Management and Prevention (GINA) criteria. Samples were 2-8 years old children who were referred to the Motahhari children polyclinic and asthma, allergy pediatric center. Two controls (n=401) were selected for each case and they were matched for age and gender using the frequency matching method. To reduce socioeconomic difference and other unknown confounding factors, controls were chosen from area of the city in which the cases were living. Meanwhile, Hospitalized controls were selected from other patient without any allergic or respiratory disease and healthy controls were selected from healthy children that referred to health centers for growth monitoring.

For gathering the required data, the questionnaire prepared by the International Study of Asthma and Allergies in Childhood (ISSAC) phase three was used. It was translated to Persian language and data related to acetaminophen exposure were collected by interviewing parents/ guardians.

Children with positive answer to Acetaminophen use due to fever in the first year of life were compared to children who did not use as the reference group. For Acetaminophen consumption during the last year of study, at least one tablet in a year, once in 2 to 3 months and at least one per month categories were used and compared with children who once or less used as the reference group. Because reported frequency of Acetaminophen intake during the last year in “never use” category was less than 5% it was combined with the “at least once a year” category and new category (once or less use in the last year) was considered as reference group.

The Pearson chi - square was applied to assess effect of Acetaminophen exposure and other potential factors that affected on the association between Acetaminophen exposure and risk of childhood asthma. The univariate logistic regression was used to calculate crude ORs and 95 % confidence intervals and a multiple logistic regression was also applied to calculate adjusted ORs and 95 % confidence intervals for odds of development of asthma following Acetaminophen exposure in the first year of study (non-used as the reference) and during the last year of study (once or less use as the reference) to assess existence of dose-response relationship between Acetaminophen use and development of asthma during the last year of study. The risk of asthma following Acetaminophen use, adjusted for important risk factors in this association when they had significant effect on the risk of development asthma in multiple regression model.

The probable confounding factors such as using antibiotic, maternal smoking, history of asthma and other allergic disorders in the first degree relatives, history of breastfeeding, number of older siblings, number of younger siblings were included in the regression model. The STATA, release 10, statistical software was used for all analyses.

## Results

Acetaminophen use in the first year of study was reported by 172 (97.20%) and 390 patients’ guardians (98.73%) in case group and controls group respectively. Acetaminophen use in the first year of life in case group was similar to control groups and did not have important effect on the risk of asthma in study population( p=0.19).70 children of cases (48.28 %) and 124 controls (31.80 %) was used acetaminophen during the last year of study( p<0.001).

Maternal smoking during pregnancy was not reported at all. Overall, 96.14% (n= 199) of cases and 96.24 %( n=384) of controls had been breast – fed and didn’t have different frequency in two groups and effect of these variables were not adjusted in multiple regression analyses.

Table 1 gives the odds of increasing asthma following acetaminophen use during the last year of study before and after controlling for effect of potential confounding factors. In single regression model, Use of Acetaminophen during the last year of study was associated with increased the risk of asthma at 2-8 years children (crude OR _once per 2 to 3 month_: 3.8; 95% CI 2.1- 6.6, OR _at least one per month_: 4.2; 95% CI 2.5-7.3). (Table 1)

**Table 1:** Distribution of acetaminophen use and risk of asthma at 2 - 8 years

	Case n (%)	Control n (%)		Crude OR (95%CI)		P value		Adjusted OR (95%CI)		P value	
Acetaminophen use during the last year											
once or less use	21(14.50)	158(40.51)		reference				reference			
once per 2 to 3 month	54(37.24)	108(27.70)		3.76(2.15 - 6.60)		<0.001		3.35(1.82- 6.17)		<0.001	
at least one per month	70(48.28)	124(31.80)		4.24(2.47 - 7.29)		<0.001		4.22(2.36 - 7.55)		<0.001	
Adjusted variables				-		-					
Antibiotic use in the first year of life				-		-					
No	47(28.14)	184(47.42)		-		-		reference			
Yes	120(72)	204(52.60)		-		-		1.72(1.10 - 2.70)		0.01	
History of asthma in first degree relatives				-		-					
No	167(97.13)	368(91.77)		-		-		reference			
Yes	43(21)	33(8.23)		-		-		3.85(2.03 - 7.30)		<0.001	
History of allergic disorders in second degree relatives				-		-					
No	100(48.31)	233(58.10)		-		-		reference			
Yes	107(51.70)	168(42)		-		-		1.49(0.98 - 2.30)		0.06	
Number of older siblings				-		-					
0	135(65.53)	237(59.25)		-		-		reference			
1	56(27.18)	103(25.75)		-		-		0.65(0.17 - 0.63)		0.09	
2≤	15(7.28)	60(15.00)		-		-		0.23(0.10 - 0.52)		<0.001	
Number of younger siblings				-		-					
0	182(88.78)	303(75.94)		-		-		reference			
1≤	23(11.22)	96(24.06)		-		-		0.33(0.17 - 0.63)		0.001	

This increasing effect was not attenuated on adjustment for effect of potential factors (adjusted OR once per 2 to 3 month: 3.35; 95%CI 1.82- 6.17, P<0.001, OR at least one per month: 4.22; 95%CI 2.36-7.55, P<0.001).This results shows dose - dependent manner in this association and those patients consumed once per 2 to 3 month and at least one per month had higher odds of asthma compared with once or less use in the last year users.

## Discussion

In the present study, Acetaminophen use and risk of asthma in 2-8 years children using case-control design was investigated and this association was observed between Acetaminophen use and risk of asthma at 2- 8 years. The strengths of study were case-control design, increase of study power using 2 controls per case and selecting controls from the same area with cases that contributed to adjust for many unknown confounding factors.

To reduce interviewer bias, all of questionnaires were completed by a trained nurse and a standard GINA criteria was used to avoid the common diagnostic differences in childhood asthma. Acetaminophen use during the last year increased the risk of asthma at 2-8 years and strength of association was persistent even after adjusting for important factors in multiple regressions. Although, our research similary the Melbourne Atopy Cohort Study, majority of study population were consumed once or less acetaminophen during last year and we didn’t have patients in ‘’never use’’ category, lowe AJ et al in this study didn’t report dose - response relation and were suggested evidence against a causal association (17), our finding indicate the effect of increasing acetaminophen consumption on the risk of development asthma at dose -response manner that is important evidence following causal association.

In extended analyses of phase three ISSAC questionnaire from countries that contributed in ISSAC program, nearly 93% of Iranian children have used Acetaminophen in the first year of life (4). In the present study, Acetaminophen use in the first year of life among cases and controls were similar (97.2 % of cases versus 98.7% of controls) and the risk of asthma development in 2-8 years children was not associated with early Acetaminophen exposure. Nonpharmacologic interventions for short-term management of fever or modest pain is the use of ibuprofen or acetaminophen,18 may be possible explanation is frequent use of acetaminophen drop to reduce fewer and important its side effects following post - vaccination in Iranian children.

Association between acetaminophen exposure and increasing risk of asthma has been shown by considerable studies in populations with different asthma prevalence, different life styles and different medical behaviors for childhood disorders that accompany fever. There are some evidence about acetaminophen use and the increased risk of asthma based on Bradford Hill criteria of causation including: strength of the association , dose-response effect that present intrauterine environment, childhood and adults, consistency between different studies in worldwide different populations (2-9,12-14,16), exposure before response that has been reported in studies with acetaminophen exposure in the intrauterine environment, infancy and adulthood, biologic plausibility (to reduce glutathione - induced acetaminophen use that damage respiratory antioxidant defenses and potentially increased TH2 response) (9), specificity of the Association for acetaminophen that no reported for other nonsteroidal anti-inflammatory drugs (19,20). Even though, there are worldwide epidemiological and clinical evidences to support causal association between Acetaminophen exposure and developing asthma and other allergic disorders, and the association remains after controlling for many of the known potential factors in this association ; however no studies have been able to demonstrate a causal association and there is only one randomized clinical trial between 1991- 1993 using 84000 febrile children in boston university fever study. May be , stronger prospective studies into the long-term effects of frequent paracetamol use in childhood had determined function of different doses of exposure with paracetamol in increase risk of development asthma.

Although our study were done using asthmatic patients compared with other patients without any allergic disorders and all forms of respiratory disease and all of our analyses were adjusted for potential confounders including matching by sex, age and area and regression techniques in analyses makes it had correct results following growing positive evidence of an association between acetaminophen use and increasing risk of asthma , however further evidences including randomized control trials and longitudinal studies in different populations with various lifestyles are suggested. This enables public health systems to make evidence-based guidelines and clinical interventions to reduce excessive acetaminophen consumption following post - vaccination and other febrile disorders in pediatric medicine.
